# Comorbidity and retirement in cervical dystonia

**DOI:** 10.1007/s00415-019-09402-0

**Published:** 2019-05-31

**Authors:** Rebekka M. Ortiz, Filip Scheperjans, Tuomas Mertsalmi, Eero Pekkonen

**Affiliations:** 1 Department of Neurology, Helsinki University Hospital, University of Helsinki, Haartmaninkatu 4, 00029 Helsinki, Finland; 2 Department of Clinical Neurosciences (Neurology), University of Helsinki, Helsinki, Finland; 3 Department of Neurology, Tampere University Hospital, Tampere, Finland

**Keywords:** Dystonia, Cervical dystonia, Comorbidity, Working ability, Pension

## Abstract

**Background:**

Cervical dystonia (CD) is the most common form of dystonia. The onset of CD is usually before 60 years of age and it may cause severe functional and psychosocial impairment in everyday life. Recently non-motor symptoms have been reported to occur in CD substantially affecting the quality of life.

**Methods/patients:**

We studied comorbidities of patients with primary focal CD in Finland based on ICD-10 codes obtained from the care registry and patient records of 937 confirmed adult isolated focal CD patients between the years 2007–2016. The retirement months and diagnosis of retirement were calculated from pension registry information. The results were compared with 3746 age and gender-matched controls.

**Results:**

Most prominent comorbidities with primary focal CD were depression (14%), anxiety (7%), and back pain (11%). The retirement age was significantly younger in CD patients compared to control group controls (59.0 years, 95% CI 58.5–59.5 vs. 61.7 years, 95% CI 61.6–61.9) years, *p* < 0.001). For dystonia patients the most common diagnoses for retirement due to sickness were dystonia (51%), depression (14%), and anxiety (8%). Patients with anxiety and depression retired earlier than other dystonia patients.

**Discussion:**

Cervical dystonia considerably reduces working ability and leads to earlier retirement. Anxiety and depression are most notable comorbidities and their co-occurrence further reduces working ability. Our results suggest that more health care resources should be administered in treatment of CD to longer maintain working ability of CD patients. Further, psychiatric comorbidities should be taken into consideration in CD treatment.

## Introduction

Dystonia is a movement disorder characterized by abnormal movements or postures created by sustained or intermittent involuntary muscle contractions [1]. Cervical dystonia (CD) is the most common form of dystonia, with a prevalence of 394 persons per million in Finland [2].

The most prominent feature of CD is abnormal posture of head and tremor. Beside the motor symptoms, non-motor symptoms have also been reported to occur in CD [3, 4]. The onset of CD is usually before 60 years of age, and even though CD does not reduce life expectancy, it may cause severe functional and psychosocial impairment in everyday life [5, 6]. The impact of CD on the quality of life (QoL) is comparable to Parkinson’s disease, multiple sclerosis and stroke [7]. The severity of dystonia as well as non-motor symptoms have been shown to affect QoL and contribute to increased disability [8–11].

We studied comorbidities of patients with primary focal CD in the Finnish care registry. Moreover, the effect of CD on pension months before the age of 65 years from the provinces of Uusimaa and Pirkanmaa (total population 2,043,819) in Finland between years 2007 and 2016 was assessed.

## Methods

The patient material comprised all 16–85-year-old patients from the university hospitals of Helsinki and Tampere, who had dystonia diagnosis during the years 2007–2016. Based on the classification of dystonia [1], the diagnosis of focal isolated cervical dystonia was confirmed from patient records. For each patient four gender- and age-matched controls were assigned.

Comorbidities were assessed by retrieving all ICD-10 (International Classification of Diseases, version 2016) codes from the years 2007–2016 from the care registry of National Institute of Health and Welfare (THL) for CD patients and controls. The diagnoses with less than three visits per diagnosis were removed to reduce the amount of misclassified diagnoses [2].

The retirement data were obtained from Finnish Centre for Pensions. In Finland, the age limit for the old age pension was 63 years at the time of study period and the sickness pension could be granted to persons over 17 years old. If the working ability of the person is diminished at least 60% for more than 1 year, sickness pension can be applied. For 40% diminished working ability, part-time sickness pension may be applied. If disability is considered temporary, patient can apply for the rehabilitation allowance. The working ability is evaluated based on medical and socio-economic criteria. The treating physician evaluates patient’s medical condition and sets one main diagnosis and optionally supporting diagnoses.

The number of retirement months before age of 65 years was calculated for patients with confirmed primary focal CD and their controls. The age of 65 years was set as the limit to also cover the people working over the old-age pension limit. Furthermore, the number of retirement months was compared between age groups (under 45 years, 45–55 years and 55–65 years) types of retirement (old age pension or sickness pension, partial or full-time pension), and different causes for retirement (1–2 diagnostic codes per period).

Mann–Whitney *U* test was used to compare the number of pension months and age of retirement. Chi-square test was applied to compare the diagnoses between patients and control group. The log-rank test and Kaplan–Meier curve was used to analyze retirement age. Bonferroni correction was used to account for multiple comparisons. *p* < 0.05 was considered statistically significant. Distributions of variables were tested with Kolmogorov–Smirnov test. The statistical analysis was done using SPSS version 24.0 (SPSS Inc., Chicago, IL, USA).

## Results

From the years 2007–2016, 1013 records of 937 primary focal CD patients were screened from the university hospitals of Tampere and Helsinki (Table 1). 348 (37%) CD patients and 550 (15%) controls had been on the full-time or partial sickness pension. Altogether 784 other ICD-10 diagnoses were found for CD patients and controls. After Bonferroni correction, 13 diagnosis codes were left with significantly different occurrence between CD patients and controls (Table 2). The most prominent comorbidities were depression, anxiety, and cervical disc disorders. Other comorbidities were back pain, unspecific soft tissue disorders, essential tremor, tension neck, somatoform disorders, specific personality disorders, dental caries and abdominal and pelvic pain.

**Table 1:** All primary focal cervical dystonia patients over 16 years and retirement months before age of 65 years

	CD patient	Control	*p* values
*n* (total)	937	3746	
Age, mean ± SD	56.4 ± 10	56.4 ± 10	
Females	73.1%	73.1%	
Pension type, mean ± SD (*n*)			
Sickness pension, under 45 years	4.1 ± 20.2 (*n* = 937)	1.9 ± 16.9 (*n* = 3746)	< 0.001
Sickness pension, 45–55 years	12.6 ± 31.4 (*n* = 812)	4.1 ± 18.4 (*n* = 3252)	< 0.001
Sickness pension, 55–65 years	27.5 ± 43.8 (*n* = 606)	12.2 ± 31.8 (*n* = 2424)	< 0.001
Sickness pension, all	32.8 ± 67.2 (*n* = 937)	13.3 ± 47.1 (*n* = 3746)	< 0.001
Old age pension, all	19.6 ± 20.4 (*n* = 307)	21.3 ± 19.9 (*n* = 1210)	n.s
Partial sickness pension, under 45 years	0.6 ± 6.4 (*n* = 937)	0.1 ± 2.2 (*n* = 3746)	< 0.001
Partial sickness pension, 45–55 years	2.2 ± 12.1 (*n* = 812)	0.2 ± 3.4 (*n* = 3252)	< 0.001
Partial sickness pension, 55–65 years	3.2 ± 14.1 (*n* = 606)	1.1 ± 8.1 (*n* = 2424)	< 0.001
Partial sickness pension, all	4.6 ± 21.1 (*n* = 937)	1 ± 9 (*n* = 3746)	< 0.001
Partial old age pension, all	4.7 ± 16 (*n* = 298)	6.8 ± 19.3 (*n* = 1189)	n.s

*n.s.* not significant

**Table 2:** Comorbidities with cervical dystonia patients

Diagnosis	Patient (*n* = 937)	Control (*n* = 3746)	OR (95% CI)	*p* values (Chi-square)
Cervical disc disorders	23 (2.5%)	16 (0.4%)	5.9 (3.1–1.1)	< 0.001
Back pain	100 (10.7%)	213 (5.7%)	2 (1.5–2.5)	< 0.001
Unspecific soft tissue disorders	53 (5.7%)	94 (2.5%)	2.3 (1.7–3.3)	< 0.001
Essential tremor	44 (4.7%)	2 (0.1%)	92.2 (22.3–381.2)	< 0.001
Tension neck	19 (2%)	18 (0.5%)	4.3 (2.2–8.2)	< 0.001
Major depressive disorder, single episode	91 (9.7%)	126 (3.4%)	3.1 (2.3–4.1)	< 0.001
Major depressive disorder, recurrent	58 (6.2%)	67 (1.8%)	3.6 (2.5–5.2)	< 0.001
Depressive disorders	120 (12.8%)	166 (4.4%)	3.2 (2.5–4.1)	< 0.001
Phobic anxiety disorders	9 (1%)	4 (0.1%)	9.1 (2.8–29.5)	< 0.05
Other anxiety disorders	56 (6%)	59 (1.6%)	4 (2.7–5.8)	< 0.001
Anxiety disorders	61 (6.5%)	61 (1.6%)	4.2 (2.9–6)	< 0.001
Somatoform disorders	16 (1.7%)	12 (0.3%)	5.4 (2.5–11.5)	< 0.005
Specific personality disorders	17 (1.8%)	17 (0.5%)	4.1 (2.1–8)	< 0.05
Dental caries	134 (14.3%)	363 (9.7%)	1.6 (1.3–1.9)	< 0.05
Abdominal and pelvic pain	74 (7.9%)	148 (4%)	2.1 (1.6–2.8)	< 0.001

The *p* values are corrected with Bonferroni correction

*OR* odds ratio, *CI* confidence interval

On average, CD patients had significantly more full-time retirement months before age 65 years than controls (32.8 ± 67.2 vs. 13.3 ± 47.1 months, *p* < 0.001) although the amount of retirement months varied considerably. The average months of sickness pension were higher in all age groups in CD patients compared with control group. CD patients had also significantly more partial sickness pension months than controls (4.6 ± 21.1 vs. 1.0 ± 9.0 months, *p* < 0.001). No gender-specific differences were seen. Old-age pension months did not differ between patient and control groups (Table 1).

**Fig. 1:**
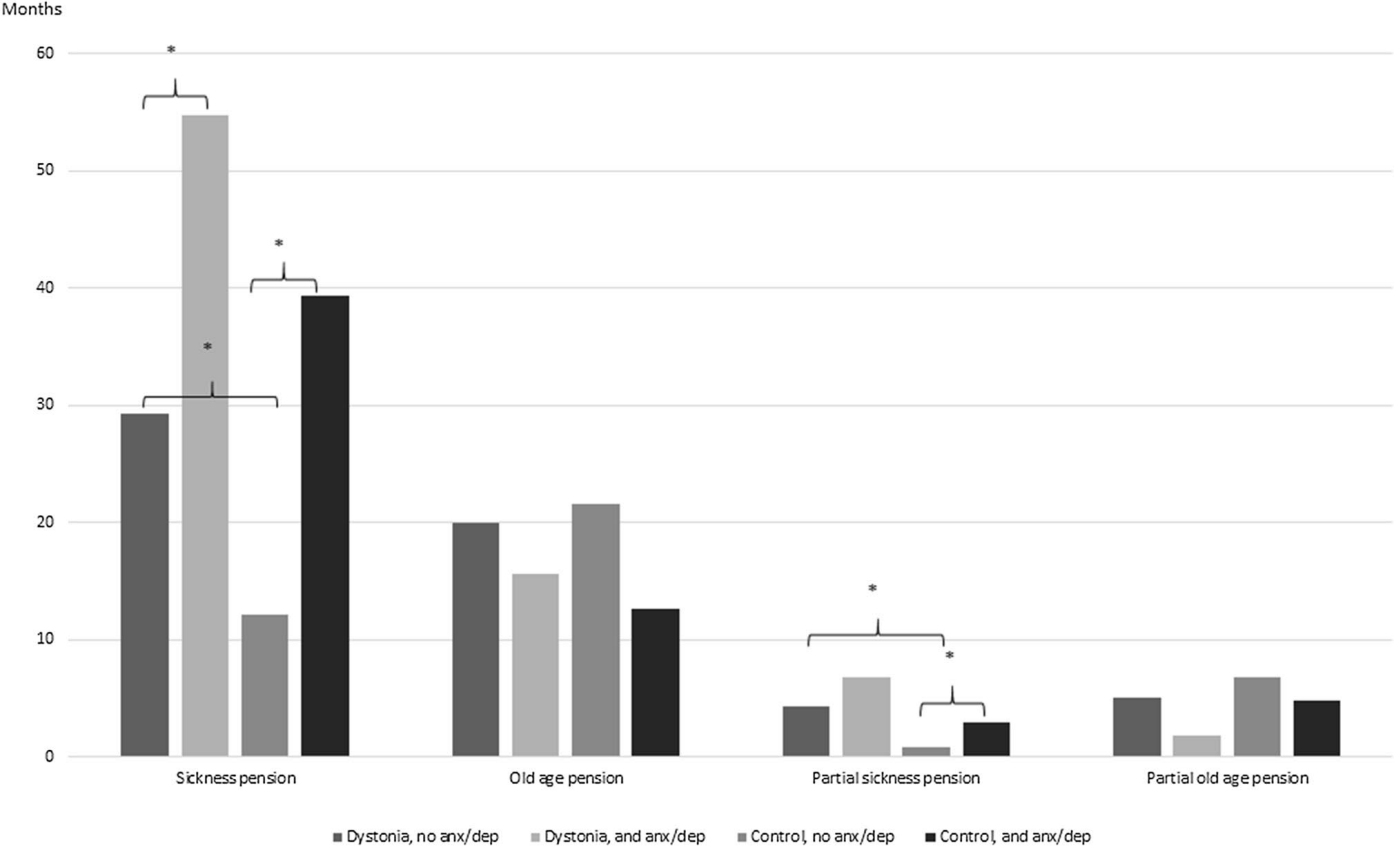
Retirement months with patients and controls with and without anxiety or depression. *p < 0.001

When the patients were divided into two groups with or without anxiety or depression (anx/dep), the patients with dystonia and anx/dep had significantly more retirement months and part-time retirement months than dystonia patients without anx/dep (54.8 ± 8.0 vs 29.3 ± 64.3 months, *p* < 0.001 and 6.8 ± 28.9 vs 4.3 ± 19.5 months, *p* < 0.001, respectively, Fig. 1). Equally, the controls with anx/dep had significantly more retirement and part-time retirement months than controls without anx/dep (39.4 ± 72.9 vs 12.1 ± 45.2 months, *p* < 0.001 and 3.0 ± 12.7 vs 0.9 ± 8.7 months, *p* < 0.001, respectively). Similarly, CD patients with anx/dep had more retirement months than control patients with anx/dep (54.8 ± 8.0 vs 39.4 ± 72.9 months, *p* < 0.001). However, the difference did not reach significance. No similar trend was seen within cervical disc disorder patients even though they had slightly more retirement months than dystonia patients without cervical disc disorder.

**Fig. 2:**
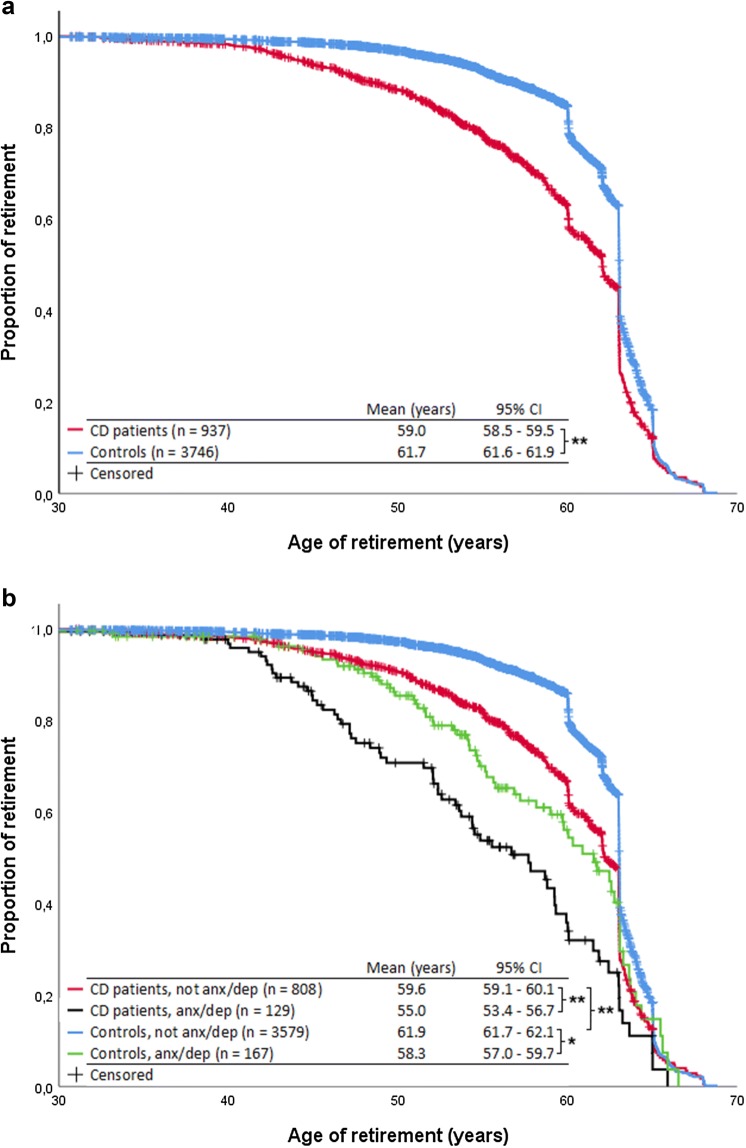
**a** The age of retirement between CD patients and controls. **b** The age of retirement between CD patients and controls when divided into groups weather or not the patients have anxiety or depression. The statistical analysis was done with log-rank test. Anx/dep anxiety or depression. *p < 0.005, **p < 0.001

The age of retirement was significantly lower with CD patients than controls (Fig. 2a). The mean retirement age for CD patients was 59.0 years and for controls 61.7 years. When CD patient and control groups were divided into groups by presence of comorbid anx/dep, CD patients with anx/dep retired significantly earlier than CD patients without anx/dep as well as control patients with anx/dep (Fig. 2b).

Primary focal CD patients had altogether 227 different diagnoses for the sickness pension. Most patients had more than one diagnosis. 15 diagnostic codes occurred more than five times with CD patients, all of them besides dystonia were either psychiatric or musculoskeletal diagnoses. Of all studied CD patients, 178 patients (19%) were retired because of dystonia. Of these, 48 patients had dystonia as only diagnosis, and another 48 patients had dystonia as the primary diagnosis of retirement. Other common diagnoses were depression (3–5%) and anxiety (2%). Compared with the ten most common retirement diagnoses in the control group, besides dystonia diagnosis, the diagnostic codes for depression and anxiety were more common in CD patients than controls. Other diagnoses had mostly similar occurrence (Table 3).

**Table 3:** Ten most common retirement diagnoses in cervical dystonia patients

Diagnosis	Patient (*n* = 937)	Control (*n* = 3746)	OR (95% CI)	*p* values
Dystonia	178 (19%)	0 (0%)	–	< 0.001
Major depressive disorder, single episode	48 (5.1%)	72 (1.9%)	2.76 (1.9–4)	< 0.001
Major depressive disorder, recurrent	26 (2.8%)	43 (1.1%)	2.46 (1.5–4)	< 0.05
Other anxiety disorders	22 (2.3%)	23 (0.6%)	3.89 (2.2–7)	< 0.05
Shoulder lesions	14 (1.5%)	25 (0.7%)	2.26 (1.2–4.4)	n.s
Intervertebral disc, thoracic, lumbar, lumbosacral	13 (1.4%)	37 (1%)	1.41 (0.7–2.7)	n.s
Specific personality disorders	12 (1.3%)	16 (0.4%)	3.02 (1.4–6.4)	n.s
Osteoarthritis of knee	11 (1.2%)	48 (1.3%)	0.92 (0.5–1.8)	n.s
Bipolar disorder	8 (0.9%)	6 (0.2%)	5.37 (1.9–15.5)	n.s
Dorsalgia	8 (0.9%)	17 (0.5%)	1.89 (0.8–4.4)	n.s

The *p* values are corrected with Bonferroni correction. In controls, within ten most common diagnoses that are not listed above and did not differ significantly are spondylosis, alcohol-related disorders, schizophrenia and polyosteoarthritis

*OR* odds ratio, *CI* confidence interval, *n.s.* not significant

## Discussion

The main finding of our study was that patients with isolated focal CD retired significantly earlier than controls. This difference was further increased if the CD was accompanied by diagnosis of anxiety or depression. The most prominent comorbidities for CD patients were depression, anxiety, and cervical disc disorders.

As in previous studies, the most prominent psychiatric comorbidities with focal CD patients were depression and anxiety [12, 13]. Also, the amount of somatoform disorders and personality disorders was higher with CD patients. However, the occurrence of psychiatric symptoms was smaller than in previous studies, and 13% of CD patients had depression and 7% had anxiety, odds ratios being 3.2 and 4.2, respectively. In prior studies, 50–91% of screened CD patients have had risk of any psychiatric diagnosis, while in general population the risk has been 35% [13, 14]. The smaller amount of psychiatric diagnoses is explainable by methodological difference. As we did not actively screen for psychiatric symptoms, it is likely, that the true comorbidity is higher, and a considerable proportion of psychiatric disorders is not diagnosed.

The incidence of psychopathology does rise with other chronic illnesses, and when dystonia has been compared with other chronic illnesses, the differences in the occurrence of psychiatric comorbidities have not been as pronounced [14]. However, when compared with another chronic illness (alopecia), the risk of developing a psychiatric symptom was still increased with CD patients [14]. Moreover, the onset of psychiatric symptoms has been reported to precede motor symptoms [13, 15, 16], suggesting that psychiatric symptoms are not merely reactions to difficulties created by the motor aspects of CD [14].

Of the other non-motor symptoms, pain is more common in CD than in other dystonia. The severity of CD has been reported to correlate with pain, and BoNT treatment alleviates pain [17]. On the other hand, central mechanisms of pain in CD have been hypothesized, and the threshold of pain perception is decreased [18, 19]. Pain has also high correlation with depression in dystonia [12]. 50% of CD patients complained of headache and 10–20% of the patients had chronic headache [20]. In our cohort, 2.1% of the patients were diagnosed with tension neck, and the occurrence was 4.2-fold compared with controls. The possible co-occurrence of the headache attributed to CD was not distinguished. Also, the unspecific code of other soft tissue disorders (M79) including limb pain, rheumatism and fibromyalgia was elevated twofold suggesting increased pain sensation. Moreover, the occurrence of abdominal pain was twice as common in CD group, but no increase of other gastrointestinal disorders was seen. Abdominal pain has been associated with anxiety and depression [21], and it is possible, that with CD patients, the psychiatric conditions increase the perception of abdominal pain.

The slight elevation of dental caries with CD patients might be the reflection of the motor limitations in CD affecting dental hygiene. However, CD patients could also have more dental evaluations because of orofacial manifestations of dystonia.

The occurrence of cervical disc disorders was elevated in CD group 5.7-fold compared with controls. CD has previously been associated with radiculopathies, spondylosis, and myelopathies as complications [22–25]. The repetitive dystonic movements and posture lead to increased stress on the cervical spine. This together with age-related degenerative changes is considered to be an etiology of degenerative spinal processes and eventually cord compression. In our cohort, no significant number of myelopathies were observed with primary focal CD patients. However, the definition of primary focal CD itself affected the selection of patients and some myelopathy patients might have been left out of analysis.

CD was also associated with the essential tremor (ET) in our study. However, the misdiagnosis of ET is common [26, 27]. The tremor associated with dystonia has been reported being of different neurophysiological etiology than ET, but clinical differentiation might be difficult [28, 29]. Thus, true comorbidity cannot be determined from this result.

The employment and working efficacy of dystonia patients has shown to be reduced and CD patients were shown to retire earlier in a Finnish questionnaire-based study [30–32]. Employment is also affected by CD and 69% of the patients were reported to have reduced overall productivity in a study by Molho et al. [31]. In another study by Molho et al. [32], CD had negative impact on employment status and CD-associated pain was one significant contributing factor. In our study, CD patients had in average 28 sickness-related pension months more than control subjects. The difference was seen in all studied age groups. Similarly, the number of partial sickness pension months was increased. Moreover, the age of retirement was 3.7 years younger with CD patients than controls. The results suggest that CD affects considerably working ability.

Most common retirement diagnoses after dystonia were depression and anxiety. Also, CD patients retired more often because of depression and anxiety than controls. In other studies, comorbid anxiety and depression, have been reported to reduce QoL independent of the motor symptoms of CD [8, 9]. In our cohort, the subgroup of patients with anxiety or depression had significantly more sickness-related retirement months than dystonia patients without anxiety or depression. Similarly, dystonia patients with anxiety or depression retired earlier compared to controls with anxiety or depression. The results imply, that comorbid psychiatric disorders reduce working ability further and their screening and treatment should be taken into consideration. Moreover, targeted interventions to address psychiatric comorbidities in CD could improve psychosocial wellbeing and QoL [10].

The reasons for earlier retirement were not assessed in this study. Previously, no clear work-related factors were found to be associated with earlier retirement [30, 31]. However, the burden of CD on everyday life, including severity of motor symptoms and pain causes reduced productivity and it is likely that it is an important factor affecting earlier retirement [32]. The patient material covered almost all CD patients treated in Uusimaa and Pirkanmaa provinces and the obtained information on pension status was complete creating a reliable source of information.

The study material was collected from the university hospitals of Helsinki and Tampere covering mostly the provinces of Uusimaa and Pirkanmaa in Southern Finland. This might affect study results as the retirement age in our control group (61.7 years) was higher than the retirement age in the Finnish population, which varied from 58.4 to 60.7 years in 2007–2016 [33]. The percentage of working age population receiving retirement was 11.5% in Finland, while in Uusimaa the percentage was lower (8.7%) and in Pirkanmaa the average (11.3%) [34].

Limitations of this study were that as a registry study no patients were contacted, and the data are based on available registry information and possible unemployment or studying could not be taken into consideration. Moreover, the comorbidity diagnoses were not actively screened, so results may underestimate true prevalence. The reliability of diagnoses from care registries varies, and is at the best very good, but the obtained information, being of secondary nature, is at the best directional [35].

## Conclusions

Cervical dystonia considerably reduces working ability and leads to earlier retirement. Anxiety and depression are most notable comorbidities and their co-occurrence further reduces working ability. Our results suggest that more health care resources addressing psychiatric comorbidities should be devoted to the treatment of CD to maintain the working ability of CD patients. Further, non-motor symptoms, particularly psychiatric comorbidities should be more actively screened and taken into consideration in CD treatment.
